# For the physiology of cooperative breeding, it's time to move beyond stress: A Comment on: ‘Stress in an underground empire’ (2022) by Medger

**DOI:** 10.1098/rsbl.2022.0375

**Published:** 2022-10-05

**Authors:** Phoebe D. Edwards

**Affiliations:** Department of Psychology, University of Toronto Mississauga, Mississauga, Ontario, Canada L5L 1C6

In cooperatively breeding species, socially dominant individuals monopolize reproduction, while subordinate individuals experience anything from lower rates of reproductive success to total suppression of reproductive function. The proximate, physiological mechanisms that allow for such reproductive suppression are of great interest. In this respect, the social mole-rats (subterranean rodents in the family Bathyergidae) have proven to be exciting species for studying the evolution and maintenance of cooperative breeding in vertebrates. The most extreme of these species is the naked mole-rat (*Heterocephalus glaber*), which lives in large colonies of up to hundreds of individuals. Breeding is monopolized by a single female called ‘the queen’ and a male breeder, with all other colony members suppressed from reproduction [1,2]. This reproductive suppression is so extreme that the non-breeding colony females—called the ‘subordinates’—are typically anovulatory [3]. Since the 1990s, it has been hypothesized that social stress in the subordinates, due to aggression from the queen, results in chronic elevation of glucocorticoids, thereby inhibiting reproductive function [4]. However, early tests of that hypothesis [4–6], and our recent work [7], have failed to find evidence that this is the case. Similarly, in cooperatively breeding Damaraland mole-rats (*Fukomys damarensis*), dominant-imposed stress has not been found to be important in subordinate reproductive suppression. Glucocorticoid concentrations do not differ between dominant breeders and subordinates, and subordinate individuals do not reproduce even if the dominants are removed from the group, likely owing to within-colony inbreeding avoidance [8].

However, in a recent *Biology Letters* review [9], Medger proposes that dominant-imposed stress, as reflected by elevated glucocorticoid concentrations in subordinates, may yet play a role in reproductive suppression in these species. The author hypothesizes that in times of colony instability, aggression by the dominant female toward the subordinates may increase, thereby elevating subordinate glucocorticoid concentrations and imposing reproductive suppression. These effects of colony instability would be rare to observe opportunistically in captive colonies, and so they propose that this may be more common in field environments with natural seasonal variation. The author concludes by suggesting the collection of further data to substantiate this hypothesis.

While this review provides interesting and thought-provoking ideas, I raise several counterpoints to the author's interpretation and provide an alternative hypothesis. First, I propose that the colony instability and seasonal data discussed in the review do not actually support the hypothesis that glucocorticoids suppress reproduction in subordinates in these instances. I provide evidence for an alternative interpretation: that elevated subordinate glucocorticoid concentrations during colony instability and seasonal changes are a reflection of increased ovarian activity, not social stress. Second, while I agree that field studies are important in understanding the natural biology of any species, I contest that even if such a relationship were to be found in the field, it has little bearing on the overall proximate mechanism of reproductive suppression in these cooperative species. Finally, I outline that the major hole in our understanding of reproductive suppression in cooperative breeders is how cues from the social environment are translated to the reproductive axis in the absence of glucocorticoid action. I argue that our collective efforts may be better spent on finding these novel neurobiological mechanisms rather than searching for a significant role for glucocorticoids, which have been rejected time and time again as a primary mechanism for subordinate reproductive suppression in cooperative breeders (reviewed in [10,11]).

To emphasize how the glucocorticoid and reproductive suppression hypothesis in these cooperative mole-rats is heavily rooted in dogma more than actual data, foundational papers in the field of endocrinology must be highlighted. Prominent reviews in the early 2000s describe a negative, suppressive relationship between glucocorticoids and reproductive function, and anything that deviates from this is the exception to the rule [12–14]. These three reviews have collectively been cited 11 711 times (Google Scholar, 14 August 2022). However, complicating this seemingly direct relationship is evidence that glucocorticoids also promote several reproductive processes. Many in our field (myself included) are guilty of colloquially referring to glucocorticoids as ‘stress hormones' but this can be a problematic oversimplification [15]. Glucocorticoids are fundamentally metabolic hormones that are critical for many life processes and influence the expression of thousands of genes [15]. They increase around ovulation in several mammalian species [16]. They are elevated in most mammals during late pregnancy [17] and are essential for normal fetal development [18,19]. There are examples of species where reproductive males have elevated glucocorticoids during the breeding season, and this may be to facilitate processes like territorial guarding and intra-sexual competition, rather than as a consequence of those behaviors [20,21].

Bearing this in mind, I first discuss the evidence that Medger [9] suggests supports the stress and reproductive suppression hypothesis during colony instability in naked mole-rats. The crux of her argument is that stress-induced reproductive suppression should kick in during instances of colony instability when potential usurper females begin to reproductively activate to become the new queen, and must be suppressed by the queen's aggressive attacks. Medger acknowledges that no relationship has previously been detected between levels of queen aggression and subordinate glucocorticoid concentrations [22]. However, she points toward indirect evidence from a laboratory study where several individuals were removed from their colonies, creating a destabilization event [23]. The remaining colony females displayed both elevated progesterone concentrations (signalling reproductive activation) and elevated cortisol concentrations [23]. Medger interprets this as the colony females attempting to become breeders (elevated progesterone), and then their subsequent punishment by the queen (elevated cortisol), thereby re-establishing suppression.

However, female naked mole-rats that are removed from the colony (and hence reproductively activate) and housed alone *also* show increased glucocorticoid concentrations [4,7,23]. This indicates that the glucocorticoid increase is not related to receiving aggression from the queen. Rather, it is potentially associated with the onset of reproductive maturation. A comparable relationship has been seen in cooperatively breeding common marmosets (*Callithrix jacchus*), where females removed from the group and paired with a male partner have elevated glucocorticoid concentrations [24]. Studies in humans [25], laboratory rats [26,27], sheep [28] and elephants [29] have shown that glucocorticoids are elevated at ovulation or just prior to ovulation (proestrus). Hence, it is probable that the elevated glucocorticoid concentrations in naked mole-rats that are becoming reproductively active are related to increasing ovarian function. Similarly, Medger [9] discusses a study in Damaraland mole-rats where subordinate females have elevated glucocorticoid concentrations in the wet season, which is a time of higher reproductive activity for this species [30]. Though the author mentions that there are multiple possible explanations for this seasonal difference (including increased reproductive effort) she suggests that it may be an effect of increased dominant aggression causing glucocorticoid-induced suppression during this time. I again emphasize the positive association between glucocorticoid concentrations and potential subordinate reproductive activity.

I disagree that the present evidence indicates subordinate suppression by glucocorticoids in these mole-rat species during colony instability or seasonal changes. But, even if the hypothesized mechanism does exist, I ask what this means for the central question of how these cooperative breeders are suppressed at all other times? These two species have been studied in captivity for decades, and subordinates are reproductively suppressed in laboratory colonies without elevated glucocorticoids [4–8]. If the mechanism proposed by Medger [9] is centrally important, these artificially stable, captive colonies should lack reproductive suppression. Because this is not the case, it is unlikely that glucocorticoids play a necessary or essential role in the maintenance of reproductive suppression.

If glucocorticoids do not play a central role in subordinate reproductive suppression in cooperative breeders, then the major unanswered question is how, mechanistically, are the conditions of the social environment communicated to the reproductive axis? There are some promising neurobiological mechanisms for understanding this. For example, gonadotropin inhibitory hormone (GnIH), or RFamide-related peptide-3 (RFRP-3) in mammals, is a neuropeptide that is generally a negative regulator of the reproductive axis (reviewed in [31–33]). In naked mole-rats, subordinates display increased RFRP-3 immunoreactivity in important reproductive centres of the brain [34]. Work in other species has shown that RFRP-3 neurons have glucocorticoid and corticotropin-releasing hormone receptors and thus can be influenced by the stress axis (as mentioned by Medger [9]). But, they also have adrenergic receptors, serotonergic receptors, and oestrogen receptors, among others [31–33]. Studies in birds have shown that GnIH responds to conditions in the social environment that are unrelated to social stress, including visual exposure to opposite-sex conspecifics (thought to be facilitated by norepinephrine [35]) and access to nesting sites [36]. This is only one example to illustrate how many pathways may communicate to the reproductive axis without the need for signalling by glucocorticoids. There are many more candidate mechanisms involved in social reproductive suppression, and mechanisms may differ across species (reviewed in [37]).

Much can be learned about the natural social dynamics of different species from continued fieldwork, and hormone sampling in the field is a good tool for answering certain questions. However, I contend that in naked mole-rats and Damaraland mole-rats, the most important future work in understanding the proximate mechanism of reproductive suppression will be intensive neurobiological and molecular studies. These species are important model systems for answering this question because their reproductive suppression is so extreme and because they can be kept in the laboratory more readily than other cooperative breeders (e.g. social carnivores). Thus, continued neurobiological and molecular work in the social mole-rats should shed light on mechanisms of reproductive suppression, with potential applications to the maintenance of cooperative breeding in general.

## Data Availability

This article has no additional data.
